# Relationship among moral authority and emotional empowerment: perspectives of clinical nurses in Imam Reza (AS) Kermanshah, 2015

**Published:** 2015

**Authors:** R Cheraghi, M Mohammadi, E Mohammadi, F Esfandnia, R Bayat, N Esfandnia, A Esfandnia

**Affiliations:** * Student Research Committee, Kermanshah University of Medical Sciences, Kermanshah, Iran

**Keywords:** moral leader, psychological empowerment, Imam Reza hospital

## Abstract

**Background.** The objective of the present research is to establish the connection among the decent masters of the emotional empowerment perspectives of nurses in Imam Reza (AS) Kermanshah Hospital in 2015.

**Methods.** The required information are gathered at the start of the towering utilization of the Internet search and library. Data relevant to the communication are gathered by using questionnaires. Standard information are gathered. The population of the research included all formal hospital-training nurses, meaning the persons responsible with the behaving of patients in Medical Sciences Kermanshah University, respectively 550. Based on the formula of the Cochran, 226 questionnaires were simple random Bayat samples; 219 surveys are delivered, used, and returned from the ultimate population. The accuracy and their confirmation are already under investigation and confirmation.

**Findings.** The findings indicated a psychological association with the ethical leadership and the enabling nurses. There is also a clear (sig = .000). Moreover, there is a direct and clear link [sig = .000] between the moral leader and enabling the psychological views of the nurses.

**Discussion.** Based on the results of the present research, it can be said that the master of morality led to the emotional empowerment of nurses. The moral evil leader of the yen means creating faith, work happiness, increased efficiency and it activates the effective organizational goals.

## Introduction

Emotional empowerment is regarded in the insight of understanding the meaning, competence, self-determination, and efficiency in the short job [**1**-**3**], while being interpreted as a mental state and intrinsic motivation. The way employees sense regarding their performance control [**1**] and the emotions arising from intrinsic motivation turn them into active members of the organization [**4**]. The concept of psychological empowerment represents the knowledge as a means of encouraging employees regarding the job requirements, their thinking, sense of duty, their level of understanding and competence, their upgrade [**5**]. The research review was made on the use of psychological empowerment, the cognitive care showing the link between the variable quality of health care [**6**] among nurses [**7**] and doctors [**8**,**9**], respectively. It is vital to consider here the amount of deaths, patient satisfaction, recovery patients, providing information to patients, privacy, and the likelihood that they will be used in evaluating the quality of patient care, the valid index not working properly [**10**]. Thus, there is a need for a coherent and integrated approach that is according to the care quality and extent, the comment on that, feeling, etc. Clinical governance is vital to realize the best possible cover [**11**]. Also, the health care management literature has a common view emerging from the lack of an emotional authority feeling, doctors being able to increase cognitive stress, absenteeism, and lower job satisfaction [**12**]. A review of literature regarding the way new leadership suggested that organizational leaders ¬instilled positive thinking was performed, by showing respect for employees, interpersonal skills, inspiration, etc., and ethics influenced subordinates follow their cause [**13**]. Due to these factors, moral leadership is direct and through clinical governance, its background creation, and promotion of mental empowerment is cognitive. Nurses, the largest human resources in most organizations and health have an important effect on the services quality [**14**] and represent the bulk of the responsibility for their care. Therefore, the importance of informed leadership on health care, especially in nursing, is clearly apparent [**15**]. A look at the style of leadership implies the existence of a variety of other styles, which are newer in terms nature and emphasis of style [**16**]. The opening leg of the third millennium was the supplier of decent authority. By definition, decent authority is “Showing a normative behavior through personal actions and interpersonal relationships and promoting this kind of behavior among the followers through bilateral communication, encouragement, and decision-making” [**17**]. The characteristics of leadership could be the following: respect for others, serving the others, just being honest, being a support in the collectivity [**18**], having developed abilities and determination in assistants [**19**], compassion, fairness, being able to advise followers to adhere and comply with ethical standards and consider the rewards and punishments for ethical and immoral behavior [**16**,**20**,**21**], respect and human relations [**22**]. In fact, leading to a variety of skills and techniques in order to meet challenges, nowadays needs change and new demands [**23**]. Research suggests that moral leadership with mental empowerment is cognitively [**24**] positive and the significant association is important. In 2014, the findings of Moghtadery and Nadi referred to the link among the ethical, emotional empowerment and career happiness and the systematic citizenship performance among the employees of private hospitals in Shiraz, showing that between the ethical characteristics [ethical climate, ethical leadership and ethical implications], emotional authority, career fulfillment, and systematic citizenship performance, there is a clear, direct link [**25**]. In 2014, the consequences of Govna Fathi et al. regarding the modeling of the link among the ethical master and the clinical governance mental authority was the following: Views of the nurse’s government hospitals in Kermanshah showed that the was a link among the ethical link and the clinical governance and mental authority from the cognitive aspect point. The cognitive relation was significant regarding the mental empowerment of clinical governance. The findings of the study indicated that the ethical leadership regarding the impact of nurses was performed directly and through a clinical governance of mental empowerment [**26**]. In 2013, Mousavi Jad’s research results with the title role of moral leadership in empowering mentality revealed that there is a clear link via the dimension empowerment between the moral leadership and items [**27**]. In 2012, the findings of Mahdad regarding the individual emotions, were similar to the ones of an association leader, Mir Jafari, about the moral and psychological health of the workplace organizational trust, showing that there was a clear and direct link among the ethical leadership and the emotional faith and the work with mental health, mental health workplace organizational trust [**28**]. Considering the above principles, the objective of the current research is to survey the link among the ethical leadership and emotional authority from the hospital nurses view in Imam Reza (AS), Kermanshah, in 2015.

## Methods 

The current research is cross-sectional and performed at 2015, in Imam Reza (AS). The information for the present research, as the first to use the library and internet search was collected. The data on population are gathered by using a questionnaire and the research was performed over the hospital fulfilment. The study sample included all registered nurses teaching hospitals (550 Nurses). Based on the formula of the Cochran, 226 questionnaires were given through a stochastic approach, and, 219 sable questionnaires were returned. In 2011, Salehniya mentioned the tool utilized for data collection: a normal survey, a questionnaire moral leadership and, according to the views of Brown et al. (2005), the model for information gathering was designed. Validity and ethical leadership reliability were reviewed and approved through the validation parameter investigation and Cronbach’s alpha value (0/88) [**21**]. The questionnaire moral leadership was in the form of ten questions and the three components of interpersonal relationships, their modeling, and pragmatism was measured. The second survey questionnaire analyzed mental abilities. In 1995, Spritzer elaborated 12 questions about the meaning and significance of four aspects, competence, self-determination impact on the range of five degrees Likert (1 = strongly disagree to strongly agree = 5) size decision. The validity and reliability study of psychological empowerment (Golparvar et. al, 2010) was done by using the confirmatory factor analysis and Cronbach’s alpha value (0/ 89) was examined and approved [**24**]. 

## Results 

According to demographic data, 58.6 percent of the partners are women and 41.4% males. When the educational distribution of the participants was examined, 17.1% had a master degree in science, 46.1% had a bachelor degree, 25.1% has a related education, and 11.7% has a regular diploma. 63.2% of the partners are married, and 36.8% were single. The distribution of participants in different age groups was the following: 50.1% among the ages of 25 and 30, 19.7% amongst 31 and 35 years, 12.5 between 35 to 41 years, 10.4% among 41-45, and 7.3% upper than 46.

**Table 1:** The link among the ethical master and the mental empowerment dimensions by using Pearson

		Moral leadership
Psychological Empowerment	R	0.867
	Sig.	0.000
	N	219
Interpersonal Relationships	R	0.861
	Sig.	0.000
	N	219
Pattern Being	R	0.852
	Sig.	0.000
	N	219
Pragmatism	R	0.835
	Sig.	0.000
	N	219

Based on the table showing the link among the ethical master and psychological empowerment of nurses by using Pearson at 95% of the shares, there is a direct link (sig = .000) and the relation factor is .867, the main theory of the current research being approved. By using Pearson at 95 percent there is a link among the ethical master and the psychological empowerment of nurses and there is also a clear direct link (sig = .000). The relation factor is no more than .8. The hypotheses of the sub-study were also approved. 

**Fig. 1:**
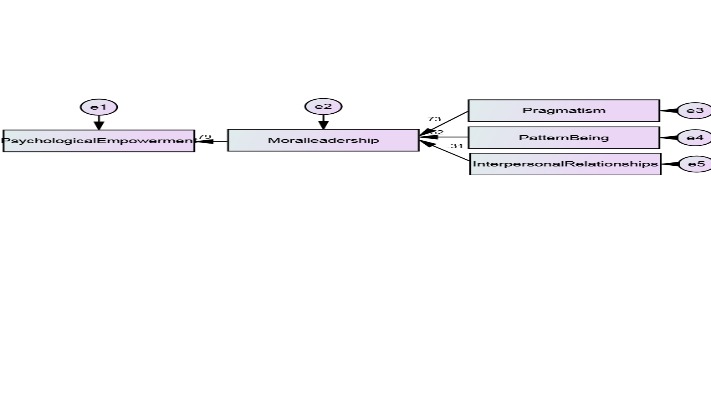
The theory of the research explained

According to the above chart, the moral leadership has a direct effect: the psychological empowerment (0/ 79), interpersonal relations (0/ 31), pattern (0/ 62) and pragmatism (0/ 86) being explained. The effects of the ethical leadership and the component “pragmatism” was of 73%. 

**Table 2:** Standardized total impacts: relationships between hypotheses

Standardized Total Effects	Interpersonal Relationships	Pattern	Pragmatism	Moral leadership
Psychological Empowerment	0.247	4.12	0.574	0.787
Standardized Direct Effects	Interpersonal Relationships	Pattern Being	Pragmatism	Moral leadership
Psychological Empowerment	0.000	0.000	0.000	0.787
Standardized Indirect Effects	Interpersonal Relationships	Pattern	Pragmatism	Moral leadership
Psychological Empowerment	0.247	0.412	0.574	0.000

According to the table 2, the general of the whole about the link among the sizes of interpersonal relationships, patterns, is pragmatic and the moral leadership rates are 0.247, 0.412, 0.574, and 0.787 respectively. The effects of the standard direct relationship between the size of interpersonal relationships, patterns, is pragmatic and the moral leadership rates are 0.000, 0.000, 0.000, and 0.787 respectively. The standardized indirect effects for the link among the sizes of interpersonal relationships, patterns, were pragmatic and the moral leadership rates were 0.247, 0.412, 0.574, and 0.000. 

**Table 3:** Final fitting: Index research model

Standard model	Acceptable level	Interpretation	The result	At reception
Chi-square CIMIN	The chi-square table	Chi-square got for a specified freedom compared	133.561	Passable
RMSEA	Younger than 05.	Fewer than 05. A proper fit	0312/ 0	Passable
TLI	Not fitted] to 1 [perfect fit]	The amount of nearly 95 a good fit	0792/ 0	Relatively acceptable
Chi-square relative CIMIN/ DF	1 - 5	Fewer than 1 shows poor fitness levels indicate the need for improvement is much than 5	2.260	Passable
PNFI		More than 5 or 6.	0523/ 0	Passable
Comparative fit index frugal PCFI		More than 5 or 6.	0525/ 0	Passable
Bentley index Bonet NFI Index Fitness Hnja Grow	Comparing the pattern via pattern without its link	Must be greater than 9.	871.	Relatively acceptable
CFI	Comparing the model to model without its relationship	Must be greater than 9.	875.	Relatively acceptable
Increase fitness index IFI	0-1	The standard rate is more than 9	876.	Relatively acceptable
Chi-square = 133.561				
Freedom degrees = 6				
Possibility level = .000				

The rate of the economics or PRATIO, a sort of Spartan proper criteria examined in it, do not realize the proper ratio, and however rather determine the amount to whatever the researcher paid the determination of free variables. The criterion extended according to degrees of freedom, model-to-model degrees of freedom could reach freedom, a rate among 0-1 and, any dimension is much less, showing that the investigator paid more expense about the free variables. Usually, higher rates of 0.5 were observed for the indicator, the ratio being of 0.600. Further, for a capacity samples amount, HOTTER guide utilized d in the current research sample, No. 41 being acceptable based on the research sample dimension of 54 companies, patterns of these indicators being also fitted. BCC, ECVA, AIC, and MECVI indicators discover the most elegant design via the smallest numbers to more sophisticated patterns examined in the research .741, 0.738,162.350, 161.561 respectively, which amounted for a MECVI of 0.738 as the enormously useful type.

## Discussion and Conclusion

This study aimed to survey the link among the ethical master and mental authority of nurses in hospitals of Imam Reza (AS). In Kermanshah, the results were highlighted by a positive link among the ethical master and mental authority of the shares, which was mental. The vital theory of the current research was approved. The ethical master and empowerment of mental significant positive correlation and secondary research hypotheses were confirmed. In 2014, the findings of the current research with the results of Govna Fathi stated the ethics and leadership according to the ethics circuit in the hospital, space, and texture to reach the efficacy of clinical governance approach, a mental empowerment feeling, promoting knowledge among nurses [**26**]. Nadi showed the powerful efforts to increase the role of private hospitals in terms of organizational citizenship behavior, the structural equation taking the psychological empowerment [**25**]. Mousavi stated that managers in hospitals and new colleagues could show a good moral behavior, interpersonal relationships, promoting efforts to reinforce such attitudes among the followers of the areas of empowerment of nurses to provide a psychological contest [**27**]. In 1931, Single et al. stated that the power from recovery leadership and the health cognitive environment increases the organizational trust [**28**]. The psychological sense of empowerment was seen as a positive approach to the characteristics of positive psychology on people and it was not unexpected that leaders were bound by moral principles, underlying the creation and promotion of emotional empowerment feelings [**26**]. In 2010, Golparvar et al. established the findings of the study direction, which were similar; their findings showed that there was a clear link between the ethical master and the mental empowerment of careers. The existence of moral leadership as the specified of infrastructure for the staff is an emotional feeling of empowerment [**29**]. The findings of the current research are in agreement via the findings of Moghtadery and Nadi (2014), Mousavi Jad (2013), Avatefi Monfared (2012), Mahdad and Mirjafari (2012). The ethical leaders believe that intrapersonal and interpersonal trust can create effects, inside and outside the organization, so that to influence the organizational success, the constant changes in technology and design of jobs and roles and duties that are required [**28**]. Considering the conclusions of the current research, it could be said that the moral leadership empowers the mental meaning that moral leadership builds trust, job satisfaction, increased efficiency of activities and goals, so that the running style and effectiveness of ethical leadership creates a positive atmosphere in the hospital. It should be noticed that the leaders feel valuable by demonstrating integrity and respect in relationships and interactions, involving employees in decision-making and showing confidence in themselves. 
